# Fabrication and Characteristic of a Double Piezoelectric Layer Acceleration Sensor Based on Li-Doped ZnO Thin Film

**DOI:** 10.3390/mi10050331

**Published:** 2019-05-17

**Authors:** Chunpeng Ai, Xiaofeng Zhao, Sen Li, Yi Li, Yinnan Bai, Dianzhong Wen

**Affiliations:** Key Laboratory of Electronics Engineering College of Heilongjiang Province, Heilongjiang University, Harbin 150006, Heilongjiang Province, China; aichunpeng@hlju.edu.cn (C.A.); zhaoxiaofeng@hlju.edu.cn (X.Z.); 2161311@s.hlju.edu.cn (S.L.); 2171256@s.hlju.edu.cn (Y.L.); 2141207@s.hlju.edu.cn (Y.B.)

**Keywords:** cantilever beam, MEMS technology, Li-doped ZnO thin film, double piezoelectric layer, acceleration sensor

## Abstract

In this paper, a double piezoelectric layer acceleration sensor based on Li-doped ZnO (LZO) thin film is presented. It is constituted by Pt/LZO/Pt/LZO/Pt/Ti functional layers and a Si cantilever beam with a proof mass. The LZO thin films were prepared by radio frequency (RF) magnetron sputtering. The composition, chemical structure, surface morphology, and thickness of the LZO thin film were analyzed. In order to study the effect of double piezoelectric layers on the sensitivity of the acceleration sensor, we designed two structural models (single and double piezoelectric layers) and fabricated them by using micro-electro-mechanical system (MEMS) technology. The test results show that the resonance frequency of the acceleration sensor was 1363 Hz. The sensitivity of the double piezoelectric layer was 33.1 mV/g, which is higher than the 26.1 mV/g of single piezoelectric layer sensitivity, both at a resonance frequency of 1363 Hz.

## 1. Introduction

At present, the development of sensor technology has attracted worldwide attention and has been noted as a key technology related to the global economy and scientific and technological progress [1,2,3]. Work on sensors based on micro-electro-mechanical system (MEMS) technology has developed very rapidly, and has become a research hotspot and focus in related fields, including industry [4], agriculture [5], national defense [6], aerospace [7], transportation [8], family services [9], and other fields. As very important inertial sensors, acceleration sensors are extensively used in the military [10] and industrial [4] and commercial applications [11]. Piezoelectric acceleration sensors, also important, are widely used in many applications, such as flexible devices [11,12], structural health monitoring [13], seismic exploration [14], and biomedical products [15].

As a third-generation semiconductor with advantages, including a wide band gap [16], high transmittance [17], nontoxicity [18], and high radiation hardness [7], ZnO has a wide range of applications in gas sensors [19,20,21], nanogenerators [22,23,24,25], data storage (memory) application [26,27], and acceleration sensors [11,28,29]. Compared with other piezoelectric materials, in addition to its nontoxicity, ZnO is also compatible with the integrated circuit (IC) process [30]. Wang et al. reported a piezoelectric acceleration sensor based on MEMS technology, in which ZnO nanowires were grown on cellulose paper as a piezoelectric layer by the hydrothermal method. The substrate was two parallel cantilever beams, and a paper mass block was connected at the ends of the two cantilevers. The resonance frequency of the acceleration sensor was 84.75 Hz and the sensitivity was 16.3 mV/g [31]. Wong et al. presented large frequency bandwidth ZnO-based single cantilever beam structure acceleration sensors by using radio frequency magnetron sputtering, and the sensitivity reached 363.09 Ω/V [18]. Raaja et al. proposed a simple analytical model for deflection, voltage, and charge sensitivity of a ZnO piezoelectric accelerometer based on a cantilever beam structure applied to structural health monitoring, and validated the model under different conditions by the finite element method. Voltage sensitivity and charge sensitivity were 60 mV/g and 2.4 pC/g, respectively [13]. Based on the above research, it is proved that ZnO has a good application in the MEMS acceleration sensor, and it also reveals that ZnO has a good compatibility with the MEMS technology. Meanwhile, acceleration sensors fabricated by MEMS technology have the advantages of miniaturization, integration, and easy packaging. At the same time, the piezoelectric constant of pure ZnO is limited, and it can be enhanced by doping Li impurities [29]. In order to improve the performance of the device, a multi-piezoelectric layer structure has been reported. Zou et al. presented novel single and triaxis piezoelectric-bimorph accelerometers, which built on parylene beams with ZnO films. The unamplified sensitivities of the *x*-, *y*-, and *z*-axis were 0.93, 1.13, and 0.88 mV/g and the cross-axial sensitivity was less than 15% [32]. Nagano et al. reported a piezoelectric tunable capacitor based on double-layered AlN thin films. The results showed a leakage current of less than 5 × 10^−5^ Am^−2^ in 500 nm thick AlN films up to 30 V [33]. Kanda et al. studied an energy harvester based on multi-layer PZT (Lead zirconate titanate). The fabricated devices with the footprint of 10 mm × 10 mm was found to provide a high output power of 53.7 μW per gravitational acceleration [34]. The multilayered piezoelectric thin films structure can be equivalent to two capacitors in a series, so the output voltage can be doubled and the voltage sensitivity of the sensors can be doubled. 

In our previous work, we designed a ZnO acceleration sensor based on a single piezoelectric layer cantilever structure by doping Li atoms as impurities to increase the resistivity of the ZnO thin film, thereby enhancing its piezoelectric characteristics. The sensitivity could reach 29.48 mV/g [29], based on the above reports [32,33,34]. In order to improve the voltage sensitivity of the acceleration sensor, a series structure double piezoelectric layer acceleration sensor based on the piezoelectric effect of the Li-doped ZnO thin film was designed, fabricated, and analyzed in this study. The application target is health detection of lathe tools in digital lathes.

## 2. Basic Structure and Working Principle

### 2.1. Basic Structure

**Figure 1:**
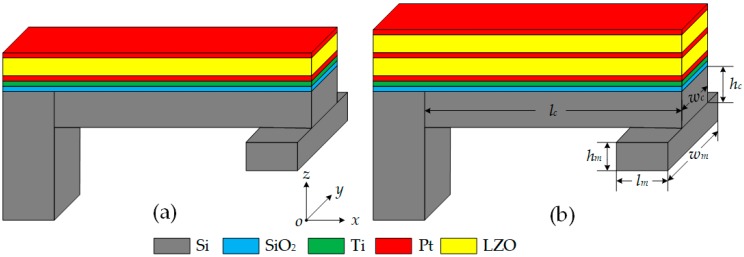
Basic structure of proposed acceleration sensor in two models: (**a**) Model I; (**b**) model II.

As shown in Figure 1, we present two structural models: Single and double piezoelectric layer structures, called model I and model II, respectively. They consist of Pt/Li-doped ZnO (LZO)/Pt/Ti thin films grown on a SiO_2_/Si cantilever beam substrate with a proof mass at the end. A Ti thin film was placed as a transition layer to ensure that the Pt and SiO_2_ layers were bonded closely. A Pt thin film was utilized as top, middle (in model II), and bottom electrodes (TE, ME, and BE). Model I is a typical single piezoelectric sandwich structure, and model II is a double piezoelectric layer structure. In model II, LZO thin films were placed among the electrodes to form a series structure in order to increase output voltage sensitivity. In Figure 1, *l_c_*, *w_c_*, and *h_c_* refer to the length, width, and height of the cantilever beam, respectively, and *l_m_*, *w_m_*, and *h_m_* are the length, width, and height of the mass block, respectively. 

The 1st mode resonant frequency of the cantilever beam can be expressed as [35,36,37]:

(1) $$f=\frac{1.875}{2\pi }\sqrt{\frac{0.236EI}{{\left(l_{c}-l_{m}/2\right)}^{3}\left(0.236\rho h_{c}w_{c}\left(l_{c}-\frac{l_{m}}{2}\right)+\rho h_{c}w_{c}\frac{l_{m}}{2}+\rho h_{m}w_{m}l_{m}\right)}}$$

where, *E* is the modulus of elasticity, *I* is the area moment of inertia about the neutral axis, *ρ* is the density of Si. It can be seen that *l_c_* is inversely proportional to the *f* frequency of the cantilever beam, and the change of *l_c_* has a greater impact on *f*.

**Figure 2:**
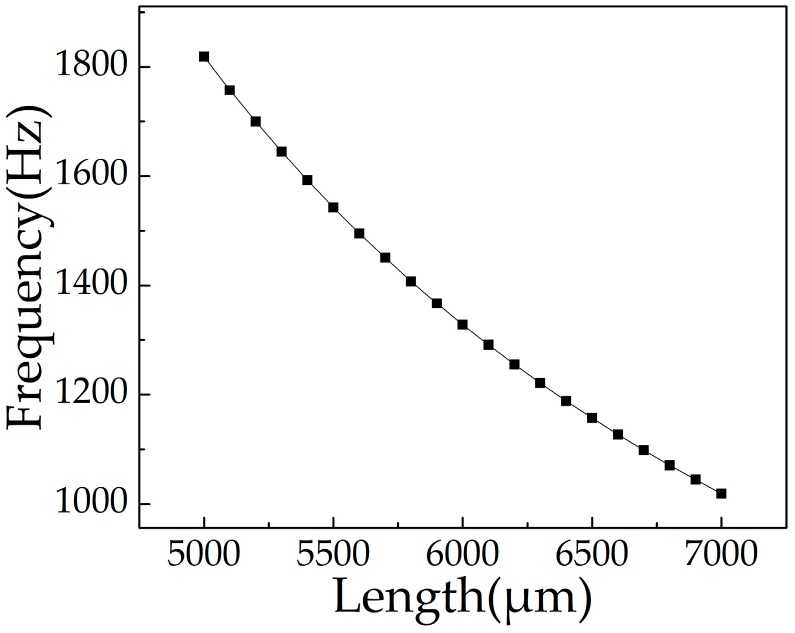
The ANSYS simulation curve between resonance frequency and length of cantilever beam.

The sensor was designed for the health detection of the lathe tool (SNR-0020R-16), the lathe tool’s resonance frequency is about 1300 Hz. On the premise of ensuring that the sensor can be installed on the lathe tool normally, meanwhile, in order to guarantee the requirement of sensitivity, we designed the *w_c_* to be 2400 μm; considering that under the action of the mass, the irreversible bending of the cantilever beam will occur when *h_c_* is too small, therefore, *h_c_* is designed to be 80 μm, and the size of proof mass is 1000 μm × 2700 μm × 395 μm. In this design, *f* is controlled by changing the value of *l_c_*. Figure 2 shows the ANSYS simulation curve of resonance frequency varying with *l_c_*. When the beam length is 6000 μm, the resonance frequency is 1327.7 Hz, which is close to that of the lathe tool. The cantilever beam is designed to be 6000 μm × 2400 μm × 80 μm in size.

### 2.2. Working Principle

**Figure 3:**
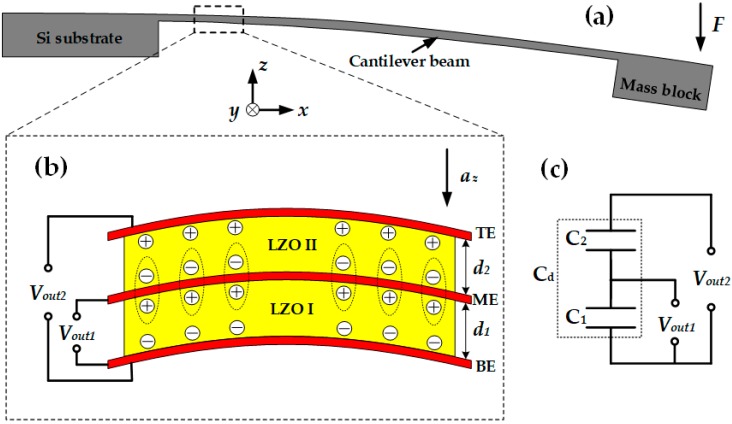
Operating principle of proposed acceleration sensor: (**a**) Deformation of cantilever beam under the action of *F*; (**b**) operating principle of double piezoelectric layer; (**c**) equivalent circuit of double piezoelectric layer.

Figure 3 shows the operating principle of the proposed acceleration sensor. With applied acceleration, the force (*F*) produced by the mass block acts on the tip of the cantilever beam, causing deformation of the cantilever beam (Figure 3a). Figure 3b shows the operating principle of the multilayer structure; *d*_1_ and *d*_2_ are the thickness of the lower and upper LZO thin films (LZO I and LZO II), respectively. Normally, without the action of external acceleration, the center of the positive and negative charges of the LZO thin film will coincide with each other, and the whole LZO thin film will be electrically neutral when external acceleration along the *z*-axis acts on the cantilever beam. The force produced by the mass block causes deformation of the cantilever beam, and the centers of the positive and negative charges are separated, which leads to positive and negative charges appearing on the top and bottom sides of the LZO thin film. Therefore, the LZO thin film can be equivalent to a capacitor. In the double piezoelectric layer structure, the charges generated by deformation on both sides of the ME are neutralized with each other and the total charge *q* remains unchanged. The double piezoelectric layer structure can be equivalent to the double capacitor series mode, which is given in Figure 3b, and the total capacitance is *C_d_*. *V*_out1_ is the output voltage between ME and BE, and the sensor works as model I. *V*_out2_ is the output voltage between TE and BE, and the sensor works as model II.

Based on the elastic mechanism analysis of the cantilever beam [29], the stress *σ* of the cantilever beam is

(2) $$\sigma =\frac{6F}{w_{c}h_{c}{}^{2}}l_{c}$$

According to the piezoelectric effect, the relationship between the output charge *q* and the stress *σ* is:

(3) $$\left(\begin{array}{c} q_{1}\\ q_{2}\\ q_{3} \end{array}\right)=\left(\begin{array}{ccc} d_{11} & \cdots & d_{16}\\ d_{21} & \cdots & d_{26}\\ d_{31} & \cdots & d_{36} \end{array}\right)\left(\begin{array}{c} \sigma_{1}\\ \vdots \\ \sigma_{6} \end{array}\right)$$

where *q*_1_, *q*_2_, and *q*_3_ denote the charges that appear on the surface of the LZO thin film that are perpendicular to the *x*-axis, *y*-axis, and *z*-axis, respectively; and *d*_11_ to *d*_16_, *d*_21_ to *d*_26_, and *d*_31_ to *d*_36_ are the piezoelectric coefficients in all directions. In accordance with small deflection theory, *σ*_1_, *σ*_2_, and *σ*_3_ are for normal stress, and *σ*_4_, *σ*_5_, and *σ*_6_ are for shear stress. Therefore, in this case, *σ*_2_ to *σ*_6_ was 0. Equation (3) can be simplified as:

(4) $$q_{3}=d_{31}\sigma_{1}$$

Substituting Equation (4) into Equation (2), we get the following equation:

(5) $$q_{3}=d_{31}\frac{6F}{w_{c}h_{c}{}^{2}}l_{c}$$

It can be seen in Equation (5) that *q*_3_ is proportional to *F* when *w_c_*, *h_c_*, and *L_c_* of the cantilever beam and *d*_31_ of the piezoelectric layer are constant.

The sensitivity of the sensor can be expressed as:

(6) $$S=\frac{V}{a}=\frac{q_{3}}{aC}$$

According to Newton’s Second Law:

(7) F=ma

Therefore, Equation (6) can be written as:

(8) $$S=\frac{6d_{31}ml_{c}}{w_{c}3h_{c}{}^{2}C}$$

where *m* is the mass of the mass block, *V* is the output voltage of the sensor, and *a* is acceleration.

In model I, the capacitance of the single piezoelectric layer can be expressed as:

(9) $$C_{s}=\frac{\varepsilon_{0}\varepsilon_{r}S}{d_{1}}$$

where *S* is the area of the electrode, *ε*_0_ is vacuum permittivity, and *ε_r_* is relative permittivity. 

For model II, charges that appear on the upper and lower sides of the ME will neutralize each other, and the ME can be regarded as a metal sheet inserted into the parallel plate capacitor. The dielectric constant of the metal is infinite, thus the capacitance of the double piezoelectric layer is:

(10) $$C_{d}={\frac{\varepsilon_{0}\varepsilon_{r}S}{d_{1}+d}}_{2}$$

Supposing *d*_1_ equals *d*_2_, here we have

(11) $$C_{s}=2C_{d}$$

According to Equations (8) and (11), we can define the sensitivity of the single piezoelectric layer acceleration sensor as *S_s_* and the double piezoelectric layer as *S_d_*, and their relationship is:

(12) $$S_{d}=2S_{s}$$

Based on Equation (12), under ideal conditions, theoretical analysis shows that when the thickness of two LZO thin films is equal, *S_d_* should be twice as much as *S_s_*.

## 3. Fabrication Technology 

**Figure 4:**
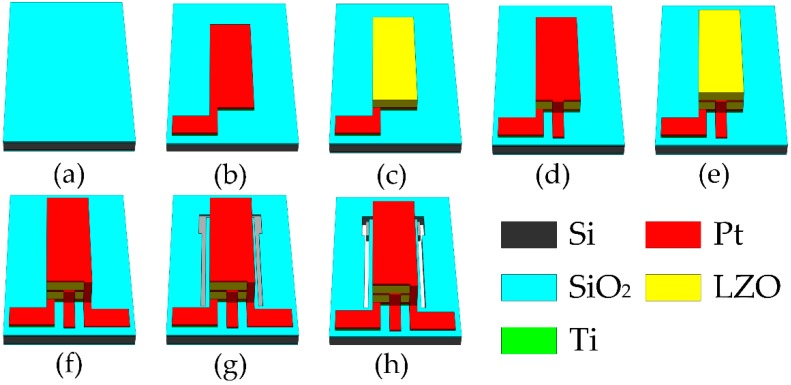
Main fabrication process of the chip: (**a**) Growing SiO_2_; (**b**) depositing Ti/Pt; (**c**) sputtering Li-doped ZnO (LZO) I; (**d**) depositing Pt; (**e**) sputtering LZO II; (**f**) depositing Pt; (**g**) etching on the top side; (**h**) releasing cantilever beam.

The chips were fabricated on n-type <100> orientation silicon wafers by MEMS technology. Figure 4 shows the main fabrication process. First, the silicon wafers were cleaned by the standard RCA (Radio Cooperation of America) process, after which SiO_2_ layers were grown on both sides of the wafers by thermal oxidation (Figure 4a). Before deposition, the chamber was pumped to a base pressure of 6.0 × 10^–4^ Pa, and a presputtering process was followed for 30 min to clean the target surface and remove any possible contamination in each sputtering process. A Ti layer was then deposited as a transition layer by direct current (DC) magnetron sputtering with 100 W power for 5 min, argon flow rate of 20 sccm (Standard-state cubic centimeter per minute), and working pressure of 1 Pa (JGP–DZS, Shenyang Sky Technology Development Co. Ltd., Shenyang, China). A Pt layer (BE) was deposited on the Ti thin film by radio frequency (RF) magnetron sputtering, sputtered for 15 min at 100 W power, with the same argon flow rate and working pressure as the Ti layer. Lift-off technology was used to enable the formation of a Ti/Pt electrode pattern (Figure 4b). A 5 wt % LZO target was adopted to prepare LZO I thin film by RF magnetron sputtering with 200 W power for 2 hours under pressure of 1 Pa, with a ratio of oxygen to argon of 5:20 sccm. The graphical process of the LZO I thin film was the same as Ti/Pt (Figure 4c). Sputtering ME (Pt), LZO II layer, and TE (Pt) were under the same conditions as above, and used the same technology for graphical processing (Figure 4d–f). After the electrodes and piezoelectric layers were fabricated, the upper surface of the silicon wafer was etched by lithography and inductively coupled plasma (ICP) etching technology to form a cantilever beam pattern (Figure 4g), and the lower surface of the silicon wafer was etched by lithography and ICP etching technology to release the cantilever beam structure (Figure 4h). In this way, the acceleration sensor based on the MEMS technology was fabricated.

**Figure 5:**
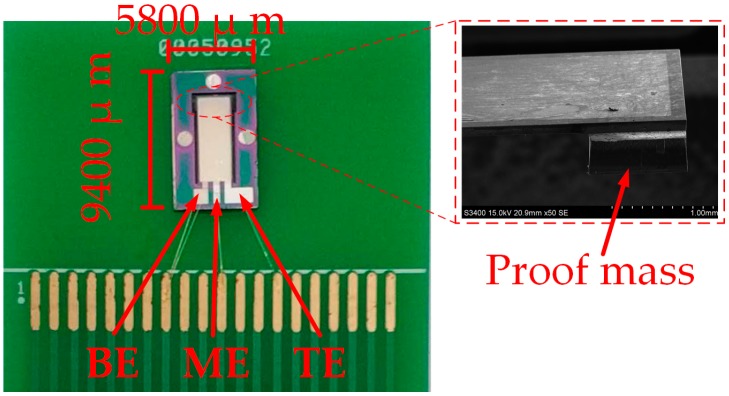
Photograph of packaged acceleration sensor chip.

As shown in Figure 5, the chip of the proposed sensor was fixed on the printed circuit board (PCB). Holes were made in the PCB below the cantilever beam to make sure the proof mass could move up and down freely when the cantilever beam was deformed. The chip size was 9400 μm × 5800 μm. Chip electrodes were connected with the PCB pads by a chip press welder (KNS4526, Kullicke & Soffa, Haifa, Israel).

## 4. Results and Discussion 

### 4.1. XRD and XPS Analysis

To determine the composition and chemical structure of the LZO thin film, XRD (AXS D8 ADVANCE, Bruker Corporation, Karlsruhe, Germany) and XPS (PHI 5700 ESCA System, Physical Electronics Co., Chanhassen, MN, USA) were used for analysis. LZO thin film samples were prepared on the Pt/Ti/SiO_2_/Si substrate with the same fabrication parameters as the chips. 

**Figure 6:**
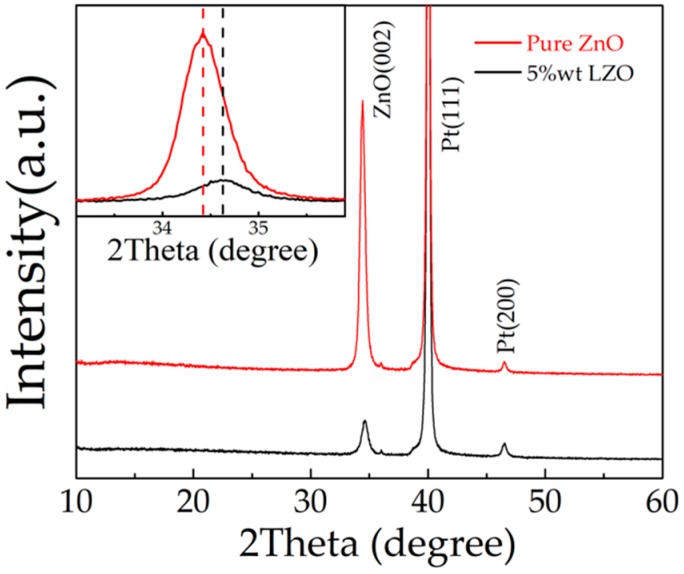
XRD spectra of the LZO thin film (inset shows (002) peaks of pure ZnO and 5 wt % LZO).

Figure 6 shows the XRD spectra of the pure ZnO and LZO thin film. The (002) peaks of ZnO, LZO, Pt (111), and Pt (200) peaks can all be observed. The (002) peak of pure ZnO is higher than that of LZO; this can be attributed to Li substituting for Zn atoms. Because of the small radius of Li atoms, the crystallinity of the ZnO thin film decreased, and reducing the peak of ZnO in 5 wt % LZO decreased. The comparison of ZnO peaks between pure ZnO and 5 wt % LZO was given in the inset. The peak of ZnO (002) of the pure ZnO sample appeared at 34.42°, and the peak of ZnO (002) of LZO shifted to the right by 0.19° to 34.61°. The substitution impurity of the small atomic radius of Li^+^ caused the decrease of lattice constant and produced compressive stress inside the LZO thin film, which led to the peak shift to the larger angle [38]. The ZnO thin film likely contained defects inside, such as V_o_ (Oxygen vacancy) and Zn_i_ (Zinc interstitial atom), which makes the ZnO films usually n-type semiconductors. After Li-doping, Li atoms as acceptor impurities increased the resistivity of the ZnO film, thereby enhancing the piezoelectric properties. 

**Figure 7:**
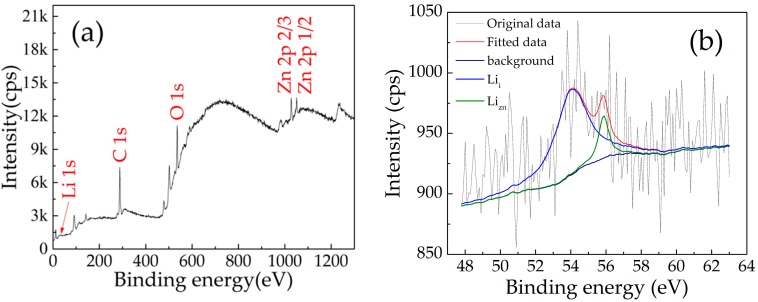
XPS spectra of the LZO thin film: (**a**) Full range; (**b**) narrow scan.

Figure 7 shows the chemical bonding states of the LZO thin film by XPS, with a scan range from 0 to 1350 eV. The binding energies were calibrated via the C 1s peak. The presence of Zn and O is confirmed in Figure 7a. The binding energy of Zn 2p3/2 and Zn 2p1/2 was found at ~1021.9 and ~1048.9. It is proved that Zn ions exist in the state of Zn^2+^ in the LZO thin film [39,40]. In the narrow scan of XPS spectra (Figure 7b), Li 1s spectra can be fitted into two different peaks, located at 54.1 and 55.8 eV, respectively. The peak at 54.1 eV was attributed to Li interstitial (Li_i_) defects corresponding to the valence state of unentire oxidation. Another Li 1s peak, located at 55.8 eV, was related to the Li-O bond, which confirms the substitution of Li^+^ at the Zn^2+^ site (Li_Zn_) [41,42,43].

This coincides with the core level of Li 1s [44,45], indicating that Li^+^ exists in the LZO thin film.

### 4.2. AFM and SEM Analysis

**Figure 8:**
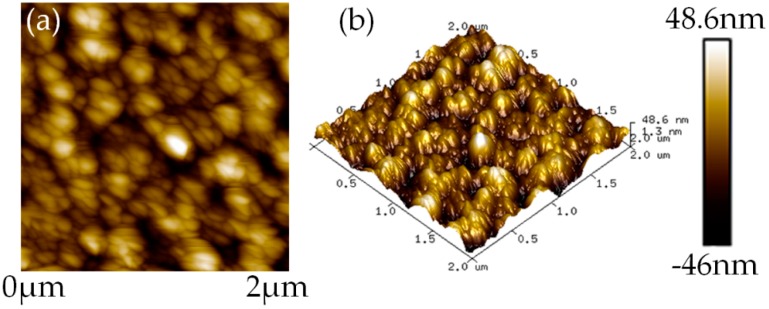
Atomic force microscopy (AFM) images of the LZO thin film: (**a**) Surface morphology; (**b**) 3D surface topography.

**Figure 9:**
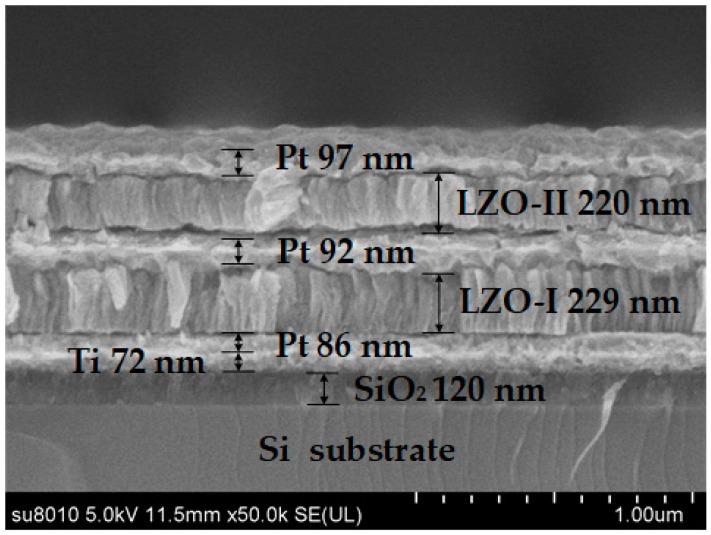
Scanning electron microscope (SEM) cross-section image of the sensor.

In order to study the surface morphology of the LZO thin film, samples were prepared as above and characterized by atomic force microscopy (AFM) (Innova, Bruker Corporation, Karlsruhe, Germany). Figure 8 shows the AFM images of the LZO thin film. The average grain size of the LZO film was about 113.7 nm and the roughness was 100 nm (all measured by AFM). A scanning electron microscope (SEM) was used to characterize a cross-section of the multilayered structure (SU8010, Hitachi, Tokyo, Japan). From Figure 9, it can be seen that the LZO film grew along the *c*-axis direction and showed a columnar structure. The particle size of the two LZO thin films coincided with the results obtained by AFM. The thicknesses of the TE, LZO II, ME, LZO I, BE, Ti layer, and SiO_2_ layer, approximately measured by SEM, are shown in Figure 9.

**Figure 10:**
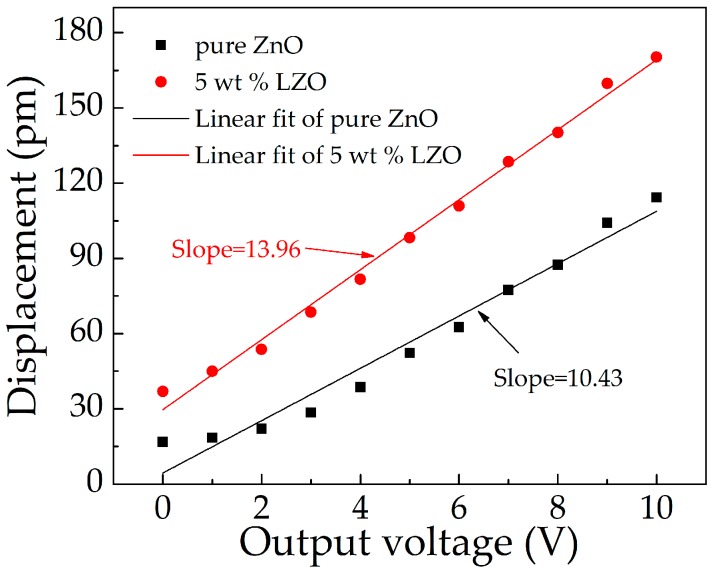
Piezoelectric characteristic of pure ZnO and 5 wt % LZO.

In order to study the piezoelectric properties of pure ZnO and LZO thin films, piezoelectric force microscopy (PFM, Bruker Multimode 8, Billerica, MA, USA) was used to measure the piezoelectric constant *d*_33_ of pure ZnO and LZO thin films. The deformation of the thin films was measured by applying voltage at both ends. As shown in Figure 10, with the increase of applied voltage in the range of 0 to 10 V, the displacement of ZnO thin films increased in the range from 16.8 pm to 114.3 pm, and that of LZO films increased in the range from 36.9 pm to 170.3 pm. The two curves were fitted linearly, and the slope of the fitted line was piezoelectric coefficient *d*_33_. We could see that the *d*_33_ of LZO thin films was 13.96 pm/V, which is higher than that of ZnO 10.43 pm/V, and the enhancement percentage is 33.84%. This proves that the piezoelectric properties of ZnO films can be improved by introducing Li as an impurity.

### 4.3. Sensitivity of the Proposed Sensor

**Figure 11:**
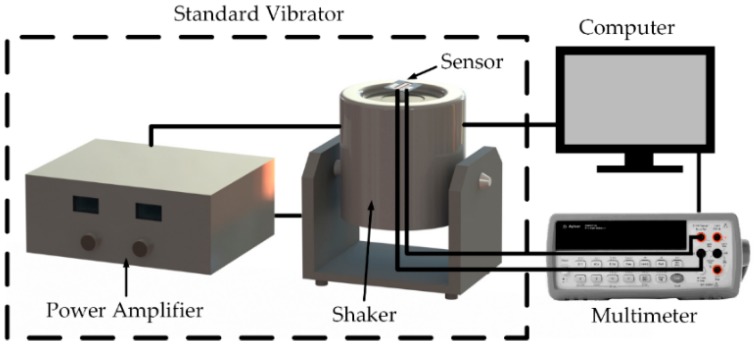
Testing system of acceleration sensor.

The testing system of the proposed acceleration sensor consisted of a standard vibrator (Dongling ESS-050, Dongling Vibration Test Instrument Co., Ltd, Suzhou, China), multimeter (Agilent 34401A, Agilent Technologies Inc., Santa Clara, CA, USA), and computer. The system can be tested for vibration and sensitivity characteristics with an exciting frequency from 50 to 20,000 Hz and acceleration from 0 to 30 g, and the test data can be automatically acquired, as shown in Figure 11.

**Figure 12:**
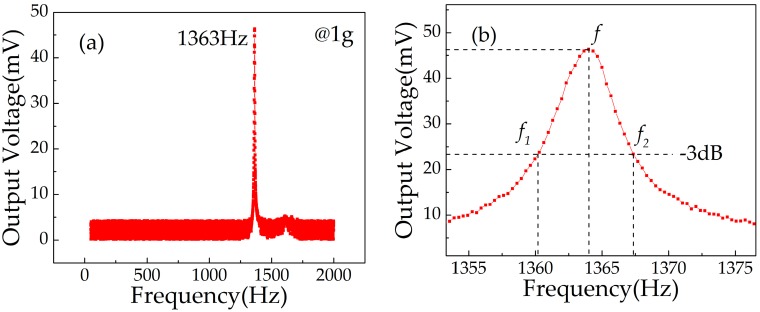
Relationship curve between output voltage and excitation frequency: (**a**) resonant frequency; (**b**) quality factor.

The response frequency characteristics of the sensor were tested by the frequency sweep mode of the standard vibrator. The sweep range of excitation frequency was from 50 to 2000 Hz under a 1 g acceleration. The acceleration sensor was rigidly fixed on the vibrator, the BE and TE of the sensor were connected to the multimeter, acceleration acted on the sensor along the *z*-axis, and the output voltage (*V*_out2_) of the sensor was collected in real time. In a certain excitation frequency range, the relationship between excitation frequency and *V*_out2_ was studied. When the applied frequency reached a certain value, the resonance effect occurred in the cantilever beam structure, and accordingly, the output voltage could reach the maximum value. Figure 12a shows the relationship curve between output voltage and excitation frequency under an acceleration of 1 g. When the excitation frequency reached 1363 Hz, *V*_out2_ reached the maximum value of 46.3 mV, and only one obvious peak was observed, which meant that only the first mode existed in the scanning frequency range. The results show that the resonance frequency of the fabricated cantilever beam structure was 1363 Hz. This is close to the simulation results of 1327.7 Hz, but there are deviations. The main reasons are as follows: (1) In the fabrication process of the cantilever beam, the uniformity of ICP etching had errors, which resulted in the uneven thickness of the cantilever beam and changed the resonance frequency; (2) although the total thickness of the multi-layer structure on the cantilever beam was much thinner than the thickness of the cantilever beam, it still affected the resonance frequency of the cantilever beam.

The quality factor *Q* represents the energy dissipated by the body in overcoming the internal friction in resonance.

(13) $$Q=2\pi \frac{E_{s}}{E_{c}}=\frac{f}{f_{1}-f_{2}}$$

where *E_s_* is the mechanical energy stored by the oscillator in the resonant state, and *E_c_* is the energy dissipated by the oscillator in the resonant state every cycle, f is the resonance frequency and *f*_1_, *f*_2_ is the half power point frequency (−3 dB). From Figure 2b, we can estimate that *Q* is 309.5.

**Figure 13:**
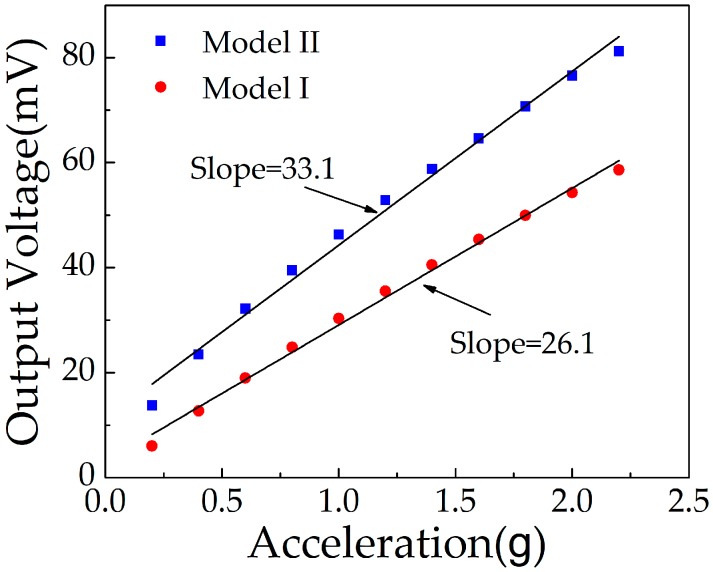
Output voltage of sensor working in model I and model II.

The output voltage characteristics of the double and single piezoelectric layers were measured by applying 0.2–2.2 g acceleration (0.2 g increment) along the *z*-axis direction of the sensor at resonance frequency (1363 Hz). As shown in Figure 13, with the increase of applied acceleration, *V*_out1_ increased from 6.05 mV to 58.59 mV, and *V*_out2_ increased from 13.8 mV to 81.2 mV. The slopes of fitting lines represent the sensitivity of the sensor. *S_d_* and *S_s_* were 33.08 mV/g and 26.05 mV/g, respectively. The sensitivity of the double LZO thin film structure was higher than that of the single LZO thin film structure, but it was not twice as large as the single LZO thin film. This is because the thickness of the LZO I film was not equal to that of the LZO II in the process of thin film preparation, so it was not consistent with the conclusion of Equation (11) in the ideal state.

**Figure 14:**
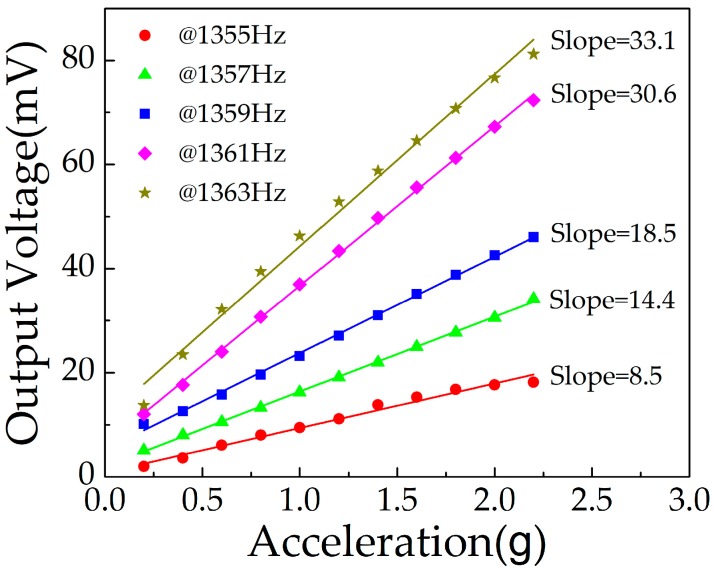
Output voltage of the sensor below resonance frequency.

**Figure 15:**
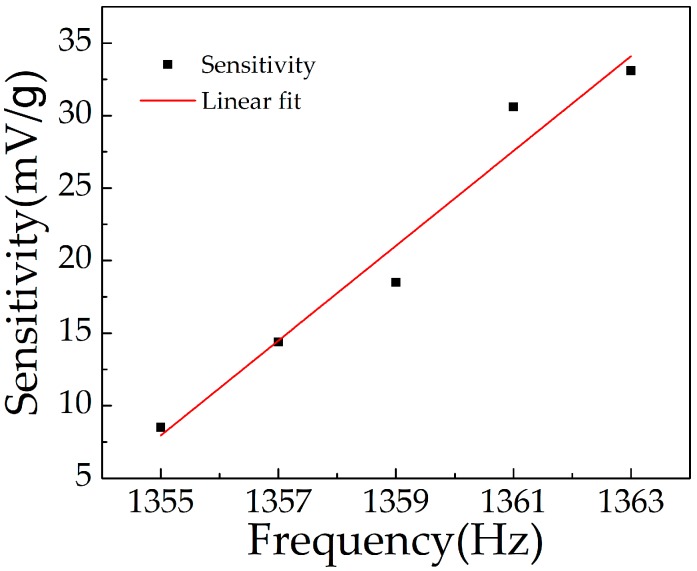
Sensitivity of the sensor in the range from 1355 Hz to 1363 Hz.

The output voltage of the sensor below the resonance frequency, in the range from 0.2 g to 2.2 g, is given in Figure 14. The sensitivity was reduced with decreased excitation frequency. Under the applied frequency of 1361 Hz, 1359 Hz, 1357 Hz, and 1355 Hz, the sensitivity was 30.6 mV/g, 18.5 mV/g, 14.4 mV/g, and 8.5 mV/g, respectively. With the decrease of excitation frequency, the resonance effect of the cantilever beam was weakened, resulting in reduced deformation. From Figure 15, we can see that the sensitivity was approximately linear with the excitation frequency, in the range from 1351 Hz to 1363 Hz.

## 5. Conclusions

In summary, this paper presents an acceleration sensor with a double piezoelectric layer structure based on the piezoelectric effect of the Li-doped ZnO thin film. Two models of single and double piezoelectric layers were established and compared. It is concluded that, in theory, the voltage sensitivity of the double piezoelectric layer should be twice that of the single piezoelectric layer. The multilayer structure was fabricated on a Si cantilever beam with a proof mass by using MEMS technology, and the ME was introduced for comparative testing. The test results show that the sensor can achieve maximum output at resonance frequency, and *S_d_* is higher than *S_s_*. It proves that the designed double piezoelectric layer structure can improve the output sensitivity of the acceleration sensor.
